# Association of Remote Patient-Reported Outcomes and Step Counts With Hospitalization or Death Among Patients With Advanced Cancer Undergoing Chemotherapy: Secondary Analysis of the PROStep Randomized Trial

**DOI:** 10.2196/51059

**Published:** 2024-05-17

**Authors:** Christopher R Manz, Emily Schriver, William J Ferrell, Joelle Williamson, Jonathan Wakim, Neda Khan, Michael Kopinsky, Mohan Balachandran, Jinbo Chen, Mitesh S Patel, Samuel U Takvorian, Lawrence N Shulman, Justin E Bekelman, Ian J Barnett, Ravi B Parikh

**Affiliations:** 1 Department of Medical Oncology Dana-Farber Cancer Institute Boston, MA United States; 2 Institute for Biomedical Informatics University of Pennsylvania Philadelphia, PA United States; 3 Penn Medicine Predictive Healthcare University of Pennsylvania Health System Philadelphia, PA United States; 4 Division of Health Policy Perelman School of Medicine University of Pennsylvania Philadelphia, PA United States; 5 Penn Center for Cancer Care Innovation Abramson Cancer Center University of Pennsylvania Philadelphia, PA United States; 6 Department of Medicine Perelman School of Medicine University of Pennsylvania Philadelphia, PA United States; 7 Center for Health Care Innovation Penn Medicine Philadelphia, PA United States; 8 Department of Biostatistics Perelman School of Medicine University of Pennsylvania Philadelphia, PA United States; 9 Ascension St. Louis, MO United States; 10 Abramson Cancer Center University of Pennsylvania Philadelphia, PA United States; 11 Corporal Michael J. Crescenz VA Medical Center Philadelphia, PA United States

**Keywords:** wearables, accelerometers, patient-reported outcomes, step counts, oncology, accelerometer, patient-generated health data, cancer, death, chemotherapy, symptoms, gastrointestinal cancer, lung cancer, monitoring, symptom burden, risk, hospitalization, mobile phone

## Abstract

**Background:**

Patients with advanced cancer undergoing chemotherapy experience significant symptoms and declines in functional status, which are associated with poor outcomes. Remote monitoring of patient-reported outcomes (PROs; symptoms) and step counts (functional status) may proactively identify patients at risk of hospitalization or death.

**Objective:**

The aim of this study is to evaluate the association of (1) longitudinal PROs with step counts and (2) PROs and step counts with hospitalization or death.

**Methods:**

The PROStep randomized trial enrolled 108 patients with advanced gastrointestinal or lung cancers undergoing cytotoxic chemotherapy at a large academic cancer center. Patients were randomized to weekly text-based monitoring of 8 PROs plus continuous step count monitoring via Fitbit (Google) versus usual care. This preplanned secondary analysis included 57 of 75 patients randomized to the intervention who had PRO and step count data. We analyzed the associations between PROs and mean daily step counts and the associations of PROs and step counts with the composite outcome of hospitalization or death using bootstrapped generalized linear models to account for longitudinal data.

**Results:**

Among 57 patients, the mean age was 57 (SD 10.9) years, 24 (42%) were female, 43 (75%) had advanced gastrointestinal cancer, 14 (25%) had advanced lung cancer, and 25 (44%) were hospitalized or died during follow-up. A 1-point weekly increase (on a 32-point scale) in aggregate PRO score was associated with 247 fewer mean daily steps (95% CI –277 to –213; *P*<.001). PROs most strongly associated with step count decline were patient-reported activity (daily step change –892), nausea score (–677), and constipation score (524). A 1-point weekly increase in aggregate PRO score was associated with 20% greater odds of hospitalization or death (adjusted odds ratio [aOR] 1.2, 95% CI 1.1-1.4; *P*=.01). PROs most strongly associated with hospitalization or death were pain (aOR 3.2, 95% CI 1.6-6.5; *P*<.001), decreased activity (aOR 3.2, 95% CI 1.4-7.1; *P*=.01), dyspnea (aOR 2.6, 95% CI 1.2-5.5; *P*=.02), and sadness (aOR 2.1, 95% CI 1.1-4.3; *P*=.03). A decrease in 1000 steps was associated with 16% greater odds of hospitalization or death (aOR 1.2, 95% CI 1.0-1.3; *P*=.03). Compared with baseline, mean daily step count decreased 7% (n=274 steps), 9% (n=351 steps), and 16% (n=667 steps) in the 3, 2, and 1 weeks before hospitalization or death, respectively.

**Conclusions:**

In this secondary analysis of a randomized trial among patients with advanced cancer, higher symptom burden and decreased step count were independently associated with and predictably worsened close to hospitalization or death. Future interventions should leverage longitudinal PRO and step count data to target interventions toward patients at risk for poor outcomes.

**Trial Registration:**

ClinicalTrials.gov NCT04616768; https://clinicaltrials.gov/study/NCT04616768

**International Registered Report Identifier (IRRID):**

RR2-10.1136/bmjopen-2021-054675

## Introduction

Patients with advanced cancer who receive chemotherapy often experience significant symptoms, declines in functional status, and hospitalization [1-3]. Patient-reported outcomes (PROs) measure symptom burden and well-being. In clinical trials, routine collection of PROs for patients with cancer undergoing treatment is associated with decreased acute care use and improved overall survival [4,5]. Similar to increased patient symptom burden, the decline in patient functional status often presages adverse events, hospitalizations, disease progression, and death [6,7]. While PROs are useful for monitoring changes in symptoms and reported activity levels, they do not provide objective measures of functional status. Step counts are a proxy measure of functional status and thus identify patients who are at a high risk of poor outcomes [6]. However, among people with advanced cancer undergoing treatment, the prognostic use of step count monitoring has never been explored [8].

Previously published studies in oncology demonstrate that lower step counts are associated with higher odds of adverse events, hospitalization, and death [6,9-11]. Yet, these studies have shortcomings that limit their generalizability to patients with advanced cancers. First, most studies were small (<50 patients), tracked step counts for less than 1 month, and focused only on patients receiving therapy with curative intent. Second, these studies measure associations between cross-sectional PROs or step counts and adverse outcomes. Few studies measured longitudinal PROs and step counts over several weeks, thus precluding evaluation of how patient-reported symptoms and objective measures of functional status interact and change over time.

Emerging value-based oncology models, including Medicare’s Enhancing Oncology Model that began in 2023, required measurement of electronic PROs. There is an urgent need to identify whether step count data complement PRO monitoring and improve early identification of patients with advanced cancer who are at risk of future adverse outcomes. The PROStep trial was a pragmatic, randomized controlled trial of patients with advanced gastrointestinal (GI) and lung cancers treated with intravenous chemotherapy [12]. Intervention patients received remote, longitudinal PRO surveys and step count monitoring. The objectives of this preplanned secondary analysis of the PROStep trial were to (1) evaluate the association between longitudinal PROs and step counts and (2) assess the association of PROs and step counts with the composite outcome of hospitalization or death. Our overarching hypothesis was that lower step counts would be associated with greater symptom burden measured by PROs and that higher symptom burden and lower step counts would be independently associated with subsequent hospitalization or death.

## Methods

### Study Design and Cohort

This is a preplanned secondary analysis of the PROStep randomized trial (ClinicalTrials.gov NCT04616768). The trial’s design, protocol, and main results have been previously published [13]. Briefly, PROStep tested the effect of clinician and patient-centered dashboards combining weekly PRO data, collected via text message, and step count monitoring, collected via a wearable accelerometer, on the primary outcomes of patient-reported clinician understanding of the patient’s symptoms and functional status. The study population consisted of 108 patients with stage IV GI or lung cancers undergoing intravenous cytotoxic chemotherapy at a tertiary academic cancer center between November 17, 2020, and June 17, 2021. Eligible patients were English-speaking, used a smartphone with SMS and Bluetooth capabilities, and received their primary oncology care at the study center. Patients undergoing monotherapy with checkpoint inhibitor therapy, targeted therapies (eg, cetuximab and trastuzumab), or oral chemotherapy without concurrent intravenous chemotherapy were excluded. Additionally, patients on active therapeutic interventional trials or confined to a wheelchair or bed were excluded.

Patients were electronically randomized in a 1:1:1 fashion, stratified by cancer type (GI or lung), to 1 of the 3 arms—(A) standard care (control), (B) PROStep intervention, or (C) PROStep intervention with active choice prompts (see Figure S1 in Multimedia Appendix 1 for CONSORT diagram). Patients randomized to the PROStep intervention received weekly 8-question text-based PRO surveys and passive step count monitoring via a wearable accelerometer (Fitbit Inspire HR; Google). PROs and step count level data were summarized in a dashboard and provided to the patient’s medical oncologist or advanced practitioner before a clinic visit. Patients in arm C also received an automated active choice text on the morning of each oncology visit, which prompted patients to discuss concerning symptoms with their oncologist.

In this preplanned secondary analysis, we evaluated the association between step counts and PROs among 57 of the 75 patients originally randomized to arms B or C who had any PRO and step count data; 18 of the 75 randomized patients did not have step counts and PRO data and could not be analyzed. As the primary analysis showed no difference in any outcome for arms B and C, study arms B and C were combined for this analysis. We then evaluated the independent associations between step count levels and PROs with subsequent hospitalization or death. The University of Pennsylvania Institutional Review Board approved the study and participants provided written or electronic informed consent during the trial.

### PRO and Step Count Assessment

Symptoms were assessed using an 8-question, text-based PRO survey drawn from the PRO version of the National Cancer Institute’s Common Terminology Criteria for Adverse Events (CTCAE), scored on a 5-point Likert scale, from 0 (not present) to 4 (disabling; see Table S1 in Multimedia Appendix 1) [14,15]. PRO surveys were sent to participants weekly on Monday mornings at 10 AM via a text message on their mobile phones. For patients who did not respond to the PRO survey, automatic reminder alerts were sent on Tuesdays and Thursdays. To measure overall symptom burden, we aggregated the scores from the 8 PRO questions in an aggregate PRO score on a 0-32 scale.

Step counts were measured using the Fitbit Inspire HR. Patients enrolled in the trial using the Way2Health app and then were given Fitbits linked to the app so that they could submit step count data by opening the app. Fitbits had a 5-day memory, so the patients received a text reminder to synchronize their Fitbit 2 times per week as well as 2 days before a clinic visit unless the data were synchronized in the prior 24 hours. The Fitbit Inspire HR measured exact daily step counts; these were summarized on a weekly basis as average daily step counts each week. Only days in which the Fitbit was appropriately synced were used in the average step count calculation (ie, days with no Fitbit data were excluded from the step count calculation).

### Outcomes

The primary outcome was a composite outcome of hospitalization or death, collected by the study research coordinator during the course of the trial for all enrolled patients. Associations between PROs and step counts were also assessed.

### Statistical Analysis

We reported descriptive patient characteristics, mean adherence to weekly PRO surveys (number of completed weekly surveys divided by the number of weeks enrolled in the study), and mean weekly adherence to step count monitoring (percentage of weeks where step counts were available for >3 days in a given week). To determine the association between PROs and step counts in a given week, we modeled their concurrent association. To determine the association of change in PRO scores and the change in step counts in a given week, we calculated bootstrapped means with 1000 iterations using bootstrapped generalized linear models. To determine the association of the longitudinal measurement of composite PRO score and average weekly steps on the composite outcome of hospitalization or death, we used bootstrapped generalized linear models with multiple “outputation” to account for repeated measures from the longitudinal data [16]. To account for the decline in the degree of association between aggregate PRO score and average weekly steps as the time between activity data and the event increases, we assumed exponential decay between the time of the PRO or step count being recorded and the composite outcome. Specifically, we multiplied regression coefficients with an exponentially decaying term and used a grid search to determine the maximum likelihood exponential decay parameter estimate used for both PRO scores and Fitbit. Finally, to evaluate the trends in PRO scores and steps in the weeks leading up to the outcomes, we computed bootstrapped means. Analyses were performed using R (version 4.2.1; R Core Team) and Python (version 3.9.13; Python Software Foundation). Two-sided hypothesis testing with α=.05 was used to assess significance.

### Ethical Considerations

This study underwent ethical review and was approved by the University of Pennsylvania Institutional Review Board (843616) and was registered on ClinicalTrials.gov (NCT04616768). Written informed consent was obtained from all participants in the trial. The study data were anonymized and deidentified. Participants in arms A, B, and C were compensated up to US $50 in gift cards upon completing their use surveys at 3 and 6 months after enrollment (US $25 each). Participants in arms B and C were permitted to keep their Fitbit as part of the trial (US $80 value).

## Results

### Baseline Characteristics

The 57 patients had a mean age of 57 (SD 10.9) years, 24 (42%) were female, 49 (86%) were White, and 3 (5%) were Black. A total of 43 (75%) patients had advanced GI cancers and 14 (25%) had advanced lung cancer (Table 1). A total of 79% (n=45) of patients completed 24 weeks of the study; the most common reasons for disenrollment were death (n=8) and voluntary drop out (n=4). Mean adherence to weekly PRO surveys was 77% (SD 29.7%), with 84% (n=48) of patients reporting PROs more than 50% of enrolled weeks. Mean weekly adherence to step count monitoring was 69% (SD 36.5%), with 70% (n=40) of patients recording step counts more than 50% of weeks enrolled in the study.

**Table 1:** Demographic characteristics of patients included in this secondary analysis of the PROStep trial. Sex and race were self-reported by patients.

Characteristics		Overall (N=57)
Age, mean (SD)		57.1 (10.9)
**Cancer group, n (%)**		
	Gastrointestinal	43 (75)
	Lung	14 (25)
**Sex, n (%)**		
	Female	24 (42)
	Male	33 (58)
**Race, n (%)**		
	Asian	3 (5)
	Black	3 (5)
	White	49 (86)
	Other	2 (4)

### Associations Between PROs and Step Counts in a Given Week

**Figure 1:**
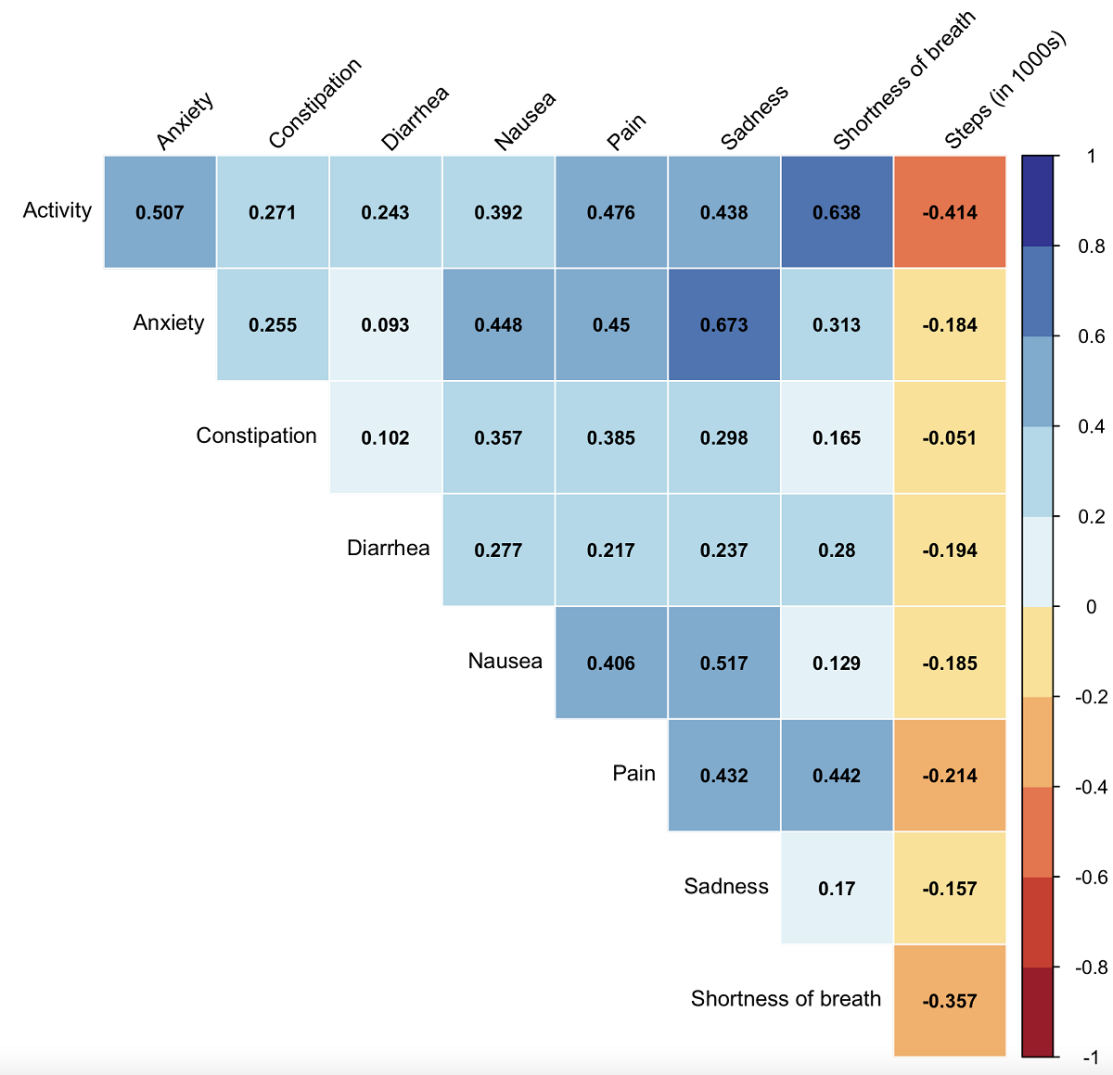
Correlations between patient-reported symptoms and step counts in a given week. Cell values represent correlation coefficients (r) between symptoms (expressed on a 0-4 scale) and step counts (expressed in thousands of steps) recorded in the same week. Blue values represent positive associations and red values represent negative associations.

In univariate analyses evaluating associations of PROs and step counts within a single week, a 1-point higher aggregate PRO score (out of 32) was associated with 150 fewer mean daily steps (95% CI –183 to –120 steps; *P*<.001) (Figure S2 in Multimedia Appendix 1). In a given week, 1-point higher scores (up to a score of 4) in shortness of breath (*P*=.03), sadness (*P*<.001), anxiety (*P*<.001), patient-reported activity (*P*<.001), nausea (*P*<.001), and diarrhea (*P*<.01) were associated with 112, 244, 125, 792, 564, and 248 fewer mean daily steps, respectively (Table S2 in Multimedia Appendix 1). Pain (–71 mean daily steps; *P*=.23), and constipation (–90; *P*=.06) were not independent factors. When examining between-PRO correlations, sadness and anxiety (*r*=0.67) and shortness of breath and patient-reported activity (*r*=0.64) were highly correlated. Patient-reported activity (*r*=–0.41) and shortness of breath (*r*=–0.36) were most correlated with decreased mean daily step count (Figure 1).

### Associations Between Longitudinal Changes in PROs and Step Counts

**Figure 2:**
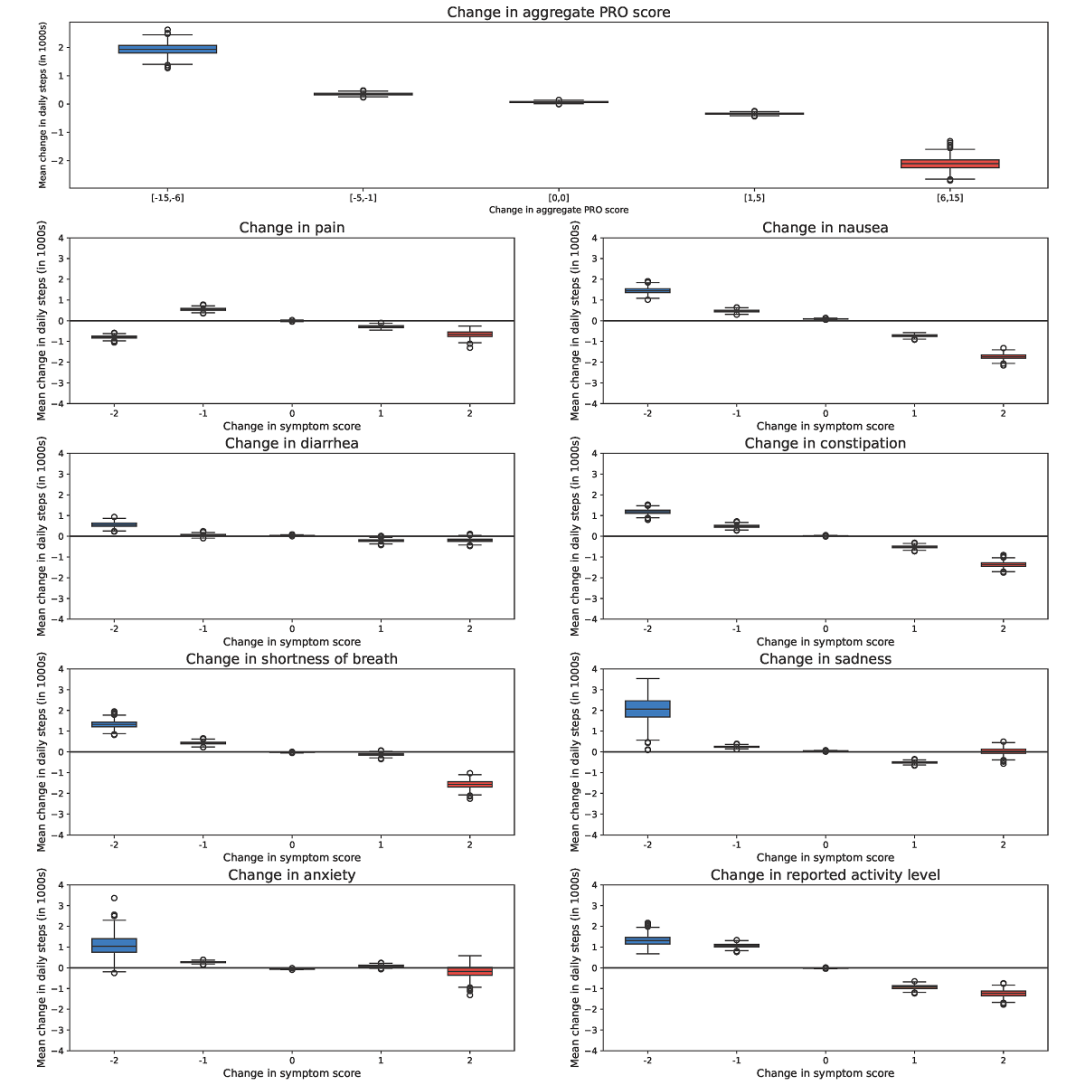
Associations between longitudinal changes in patient-reported symptoms and step counts. Longitudinal changes are displayed in box and whisker plots, measured from week n–1 to week n. Open circles represent outliers. PRO: patient-reported outcome.

In univariate analyses assessing week-to-week changes in PROs and changes in step counts, changes in aggregate PRO score from the prior week to the current week were associated with stepwise increases or decreases in the mean daily step counts (Figure 2). A 1-point increase in aggregate PRO score from the prior week was associated with a decrease of 247 mean daily steps (95% CI –277 to –213; *P*<.001). One-point increases in following PROs from the prior week had the strongest associations with decreased mean daily steps—patient-reported activity (mean daily step change –892, 95% CI –1050 to –758; *P*<.001), nausea (–677, 95% CI –770 to –588; *P*<.001), constipation (–524, 95% CI –614 to –431; *P*<.001), shortness of breath (–399, 95% CI –498 to –302; *P*<.01), pain (–304, 95% CI –409 to –204; *P*<.001), sadness (–382, 95% CI –462 to –302; *P*<.001), and anxiety (–125, 95% CI –213 to –42; *P*<.001) (Table S2 in Multimedia Appendix 1).

### Associations Between PROs, Step Counts, and Hospitalization or Death

**Figure 3:**
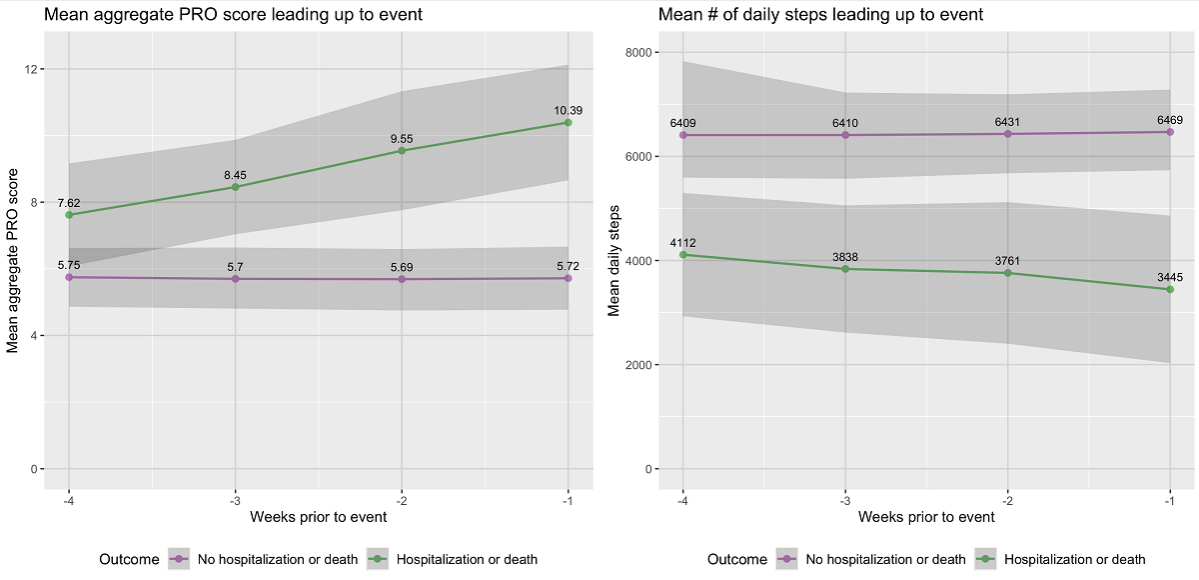
Longitudinal association between aggregate PRO symptom score, step counts, and hospitalization or death. The x-axis refers to the number of weeks before an event (hospitalization or death). Purple lines represent averages for patients without an event. Green lines represent averages for patients with an event. PRO: patient-reported outcome.

Among 57 patients, 21 (37%) patients were hospitalized and 8 (14%) died during the follow-up period. The rate of the composite outcome of hospitalization or death was 44% (n=25). On average, a 1-point increase in the aggregate PRO score was associated with a 20% increase in adjusted odds of hospitalization or death (adjusted odds ratio [aOR] 1.2, 95% CI 1.1-1.4; *P*=.01; Table 2). In a given week, a 1-point increase in patient-reported pain (aOR 3.2, 95% CI 1.6-6.5; *P*=.01), activity (aOR 3.2, 95% CI 1.4-7.1; *P*=.01), shortness of breath (aOR 2.6, 95% CI 1.2-5.5; *P*=.01), sadness (aOR 2.1, 95% CI 1.1-4.3; *P*=.03), and anxiety (aOR 2.7, 95% CI 1.3-5.6; *P*=.01) were most associated with increased odds of hospitalization or death. After adjusting for aggregate PRO score, a decrease in 1000 mean daily steps was associated with 16% increased odds of hospitalization or death (aOR 1.16, 95% CI 1.01-1.33; *P*=.03). Patients who were hospitalized or died had a progressive increase in aggregate PRO score and a decrease in mean daily steps in the 4 weeks leading up to the event, but patients who did not experience an event had no change in aggregate PRO score or mean daily steps in the prior 4 weeks (Figure 3). Compared with baseline, mean daily step count decreased 7% (n=274/4112 steps), 9% (n=351/4112 steps), and 16% (n=667/4112 steps) in the 3, 2, and 1 weeks before hospitalization or death, respectively. Mean aggregate PRO score increased by 11% (n=0.8), 25% (n=1.9), and 36% (n=2.8), in the 3, 2, and 1 weeks before hospitalization or death, respectively.

**Table 2:** Association of symptoms and step counts with the composite outcome of hospitalization or death^a^.

Symptoms^a^	Symptom		Mean daily step count (per 1000 steps)	
	aOR^b^ (95% CI)	*P* value	aOR (95% CI)	*P* value
Composite PRO^c^ score	1.20 (1.1-1.4)	.01	0.86 (0.75-0.99)	.03
Pain	3.23 (1.6-6.5)	<.001	0.84 (0.72-0.98)	.02
Activity	3.16 (1.4-7.1)	.01	0.9 (0.79-1.03)	.14
Anxiety	2.69 (1.3-5.6)	.01	0.82 (0.70-0.95)	.01
Shortness of breath	2.58 (1.2-5.5)	.02	0.87 (0.76-1.01)	.07
Sadness	2.14 (1.1-4.3)	.03	0.82 (0.65-0.95)	.01
Constipation	1.92 (0.98-3.8)	.06	0.81 (0.69-0.57)	.01
Diarrhea	1.21 (0.78-1.9)	.40	0.81 (0.69-0.94)	.01
Nausea	0.91 (0.59-1.4)	.66	0.81 (0.70-0.95)	.01

^a^Each row represents a separate model composed of the symptom in the left column and step counts (in thousands) as the predictor variables and the composite outcome of hospitalization and death as the outcome.

^b^aOR: adjusted odds ratio.

^c^PRO: patient-reported outcome.

## Discussion

### Principal Findings

In this preplanned secondary analysis of a randomized clinical trial among patients with incurable GI and lung cancers receiving chemotherapy, higher symptom burden measured by remote PROs was associated with lower daily step count. Moreover, higher symptom burden and decreased daily step counts were independently associated with an increased risk of hospitalization or death. PROs and step counts predictably worsened in the 4 weeks before hospitalization or death, whereas the individuals who did not experience hospitalization or death had stable PROs and step counts.

Prior studies among patients with cancer undergoing chemoradiation demonstrated that lower step counts, measured cross-sectionally over brief periods, are associated with subsequent acute care use and worse prognosis [6,9-11]. We build upon these findings by demonstrating that longitudinally measured PROs and step counts are predictive of hospitalization and death. PROs and step count predictably worsened in the 4 weeks before hospitalization and death. This indicates that among patients with advanced cancer receiving chemotherapy, longitudinal remotely monitored patient-generated health data may identify at-risk patients weeks before hospitalization or death—a window in which proactive interventions, including goals-of-care conversations, may improve outcomes or goal-concordant care. Importantly, decreasing patient-reported activity level was highly predictive of hospitalization or death (aOR 3.2, 95% CI 1.4-7.1) even when adjusting for step counts, suggesting that patient-reported activity may complement objective measures of step counts.

Our study is among the first to assess associations between longitudinal PROs and step counts and downstream use, among patients with cancer. We show that patients with higher symptom burden also experience fewer daily step counts in a given week. Furthermore, in longitudinal analyses, daily step counts decreased as symptom burden increased. Worsening nausea, constipation, shortness of breath, and sadness had the largest associations with decreased step count.

There is growing evidence to support routine collection of PROs, and Medicare’s Enhancing Oncology Model incentivizes PRO measurement. Our results may facilitate efforts to use these PRO data in predictive models by identifying those PROs most likely to contribute to adverse events. This study provides novel provocative data that step count monitoring independently identifies at-risk patients and could be used synergistically with PROs as part of targeted care delivery interventions to prevent acute care use or target supportive care interventions. Such interventions may include additional symptom support, home services like physical therapy, treatment modifications, palliative care consultation, or goals-of-care conversations. For instance, worsening step counts may prompt physical therapy interventions to improve functional status and perhaps reduce the risk of hospitalization. Policymakers may consider strategically incorporating activity monitoring with electronic PRO monitoring mandates to enhance risk prediction and stratification of high-risk populations. Future studies should examine how longitudinal PRO and step count data could identify decreasing chemotherapy tolerance, enabling earlier dose reduction that might improve quality of life and extend treatment tolerability. Future studies should also investigate whether longitudinal PRO and step count data predict disease progression or symptomatic disease, potentially prompting earlier imaging and switches to new therapy.

### Limitations

This study has several limitations. It includes patients with only 2 types of cancer enrolled in a clinical trial at a single tertiary cancer center and all undergoing chemotherapy, which may enrich for a sicker population that may have the most pronounced changes in symptoms and step counts. Adherence to weekly PROs and step counts among patients in this analysis was 77% (SD 29.7%) and 69% (SD 37%), respectively, which is higher than the adherence reported in real-world studies [17]. However, we excluded patients who did not have PRO or step count data, and adherence in the full cohort was similar to the levels reported in these real-world studies [12]. Moreover, step counts could only be tracked if patients wore the wearable monitor—if patients were less likely to wear the monitors in certain circumstances (eg, when symptoms worsen), data missingness may be informative. A subsequent analysis will explore how data missingness can also be used to improve predictive power. The study also used an abbreviated sample of PRO- CTCAE questions targeted to patients with GI and lung cancers; findings may differ using the full PRO-CTCAE question panel and in patients with other malignancies. Finally, our sample size and overall number of outcomes were too small to disaggregate predictors of hospitalizations or death individually. We hope future prospective work with larger sample sizes will facilitate the disaggregation of these outcomes. Nonetheless, prediction of both death and hospitalizations near the end of life can be used to trigger interventions such as goals-of-care conversations that may enable better goal-concordant care.

### Conclusions

This study of remote monitoring of PROs and step counts shows that changes in these measures predict hospitalization and death for patients with advanced cancer undergoing treatment. Future work should validate these findings in larger, more diverse populations and translate these results into interventions that can avoid acute care use and improve supportive care.

## Appendix

Multimedia Appendix 1 Consolidated Standards of Reporting Trials (CONSORT) diagram from PROStep trial; PRO survey: National Cancer Institute’s common terminology criteria for adverse events and Patient Reported Functional Status (PRFS) tool; associations between patient-reported symptoms and step counts in a given week; and association between patient-reported symptoms and mean daily step count.

## Abbreviations

aOR: adjusted odds ratio
CTCAE: Common Terminology Criteria for Adverse Events
GI: gastrointestinal
PRO: patient-reported outcome

## Data Availability

The data supporting the findings of this study are available upon request from the corresponding author. The data are not publicly available because of privacy concerns.
