# Supplementary material for: Association of Remote Patient-Reported Outcomes and Step Counts With Hospitalization or Death Among Patients With Advanced Cancer Undergoing Chemotherapy: Secondary Analysis of the PROStep Randomized Trial
Source: J Med Internet Res. 2024 May 17;26:e51059. doi: 10.2196/51059 (PMC11143393; doi:10.2196/51059)
Supplement: Multimedia Appendix 1 [file jmir_v26i1e51059_app1.docx]

Table of Contents

Figure S1. CONSORT diagram from PROStep Trial

Table S1. PRO Survey: National Cancer Institute’s Common Terminology Criteria for Adverse Events and Patient Reported Functional Status (PRFS) Tool

Figure S2. Associations between patient-reported symptoms and step counts in a given week

Table S2. Association between patient-reported symptoms and mean daily step count

| 1. In the last 7 days, how OFTEN did you have NAUSEA? | | | | |
| --- | --- | --- | --- | --- |
| Ο Never | Ο Rarely | Ο Occasionally | Ο Frequently | Ο Almost constantly |
| 2. In the last 7 days, how OFTEN did you have LOOSE OR WATERY STOOLS (DIARRHEA/DIARRHOEA)? | | | | |
| Ο Never | Ο Rarely | Ο Occasionally | Ο Frequently | Ο Almost constantly |
| 3. In the last 7 days, what was the SEVERITY of your CONSTIPATION at its WORST? | | | | |
| Ο None | Ο Mild | Ο Moderate | Ο Severe | Ο Very severe |
| 4. In the last 7 days, what was the SEVERITY of your PAIN at its WORST? | | | | |
| Ο None | Ο Mild | Ο Moderate | Ο Severe | Ο Very severe |
| 5. In the last 7 days, how much did your SHORTNESS OF BREATH INTERFERE with your usual or daily activities? | | | | |
| Ο Not at all | Ο A little bit | Ο Somewhat | Ο Quite a bit | Ο Very much |
| 6. In the last 7 days, how OFTEN did you have SAD OR UNHAPPY FEELINGS? | | | | |
| Ο Never | Ο Rarely | Ο Occasionally | Ο Frequently | Ο Almost constantly |
| 7. In the last 7 days, how OFTEN did you feel ANXIETY? | | | | |
| Ο Never | Ο Rarely | Ο Occasionally | Ο Frequently | Ο Almost constantly |
| 8. Over the past week I would generally rate my activity as: | | | | |
| 0. normal with no limitations | | | | |
| 1. not my normal self, but able to be up and about with fairly normal activities | | | | |
| 2. not feeling up to most things, but in bed or chair less than half the day | | | | |
| 3. able to do little activity & spend most of the day in bed or chair | | | | |
| 4. pretty much bedridden, rarely out of bed | | | | |


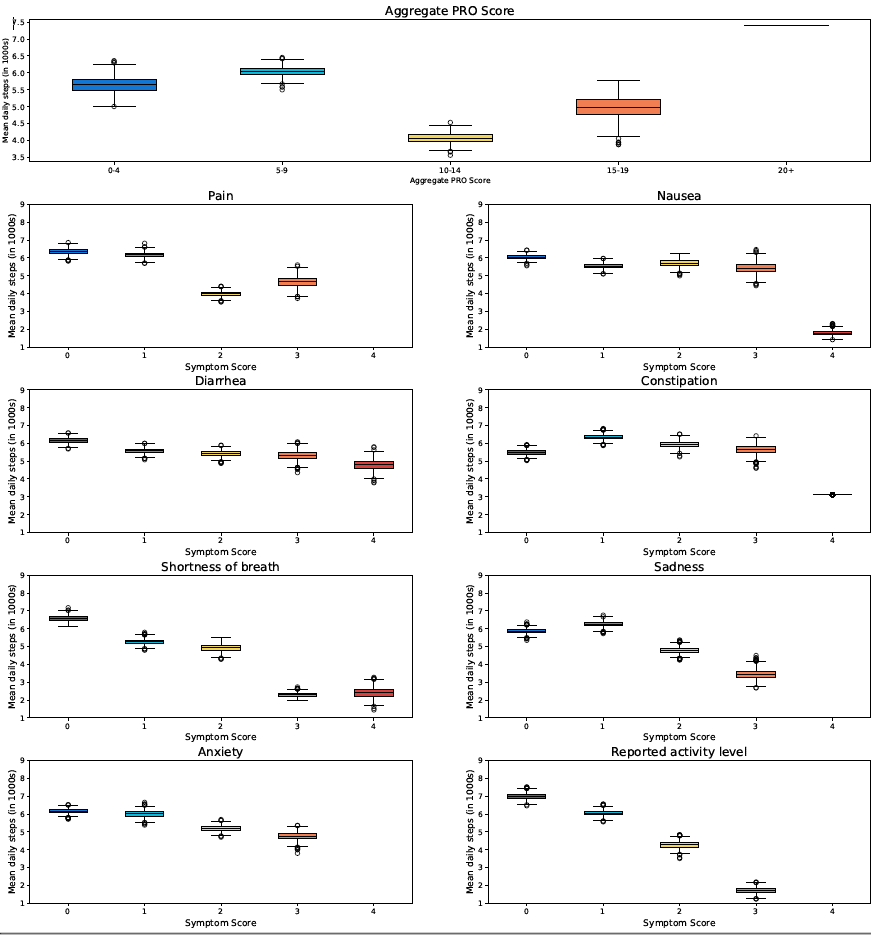


| **Patient reported outcome*^,+^**  In the last 7 days, … | **Steps*** | | | **Change in Steps^+^** | | |
| --- | --- | --- | --- | --- | --- | --- |
|  | *Steps* | *95% CI* | *p* | *Steps* | *95% CI* | *p* |
| PRO Aggregate Score (sum of the 8 below) | -150 | (-183, -120) | <0·001 | -247 | (-277, -213) | <0·001 |
| … what was the SEVERITY of your PAIN at its WORST? | -71 | (-186, 63) | 0·23 | -304 | (-409, -204) | <0·001 |
| … how OFTEN did you have NAUSEA? | -564 | (-655, -480) | <0·001 | -677 | (-770, -588) | <0·001 |
| .. how OFTEN did you have LOOSE OR WATERY STOOLS (DIARRHEA/DIARRHOEA)? | -248 | (-335, -170) | <0·001 | -137 | (-211, -73) | <0·001 |
| … what was the SEVERITY of your CONSTIPATION at its WORST? | -90 | (-185, 5) | 0·06 | -524 | (-614, -431) | <0·001 |
| … how much did your SHORTNESS OF BREATH INTERFERE with your usual or daily activities? | -112 | (-204, -15) | 0·03 | -399 | (-498, -302) | <0·001 |
| … how OFTEN did you have SAD OR UNHAPPY FEELINGS? | -244 | (-329, -150) | <0·001 | -382 | (-462, -302) | <0·001 |
| … how OFTEN did you feel ANXIETY? | -125 | (-200, -45) | <0·001 | -125 | (-213, -42) | 0·004 |
| Over the past week I would generally rate my ACTIVITY as: | -792 | (-942, -638) | <0·001 | -892 | (-1050, -758) | <0·001 |
| * Associations are between different levels of PRO score (0-4) and mean daily steps within a given week  ^+^ Associations are between change in PRO score (-4 to +4) and change in mean daily steps from the prior week to the current week  *See eTable 1 for PRO response scales.* | | | | | | |
